# IndelsRNAmute: predicting deleterious multiple point substitutions and indels mutations

**DOI:** 10.1186/s12859-022-04943-0

**Published:** 2022-10-14

**Authors:** Alexander Churkin, Yann Ponty, Danny Barash

**Affiliations:** 1 Department of Software Engineering, Sami Shamoon College of Engineering, Beersheba, Israel; 2 Laboratoire d’Informatique de l’École Polytechique (LIX CNRS UMR 7161), Ecole Polytechnique, Palaiseau, France; 3 Department of Computer Science, Ben-Gurion University, Beersheba, Israel

**Keywords:** RNA mutations prediction, RNA indels prediction, Suboptimal RNA structure

## Abstract

**Background:**

RNA deleterious point mutation prediction was previously addressed with programs such as RNAmute and MultiRNAmute. The purpose of these programs is to predict a global conformational rearrangement of the secondary structure of a functional RNA molecule, thereby disrupting its function. RNAmute was designed to deal with only single point mutations in a brute force manner, while in MultiRNAmute an efficient approach to deal with multiple point mutations was developed. The approach used in MultiRNAmute is based on the stabilization of the suboptimal RNA folding prediction solutions and/or destabilization of the optimal folding prediction solution of the wild type RNA molecule. The MultiRNAmute algorithm is significantly more efficient than the brute force approach in RNAmute, but in the case of long sequences and large m-point mutation sets the MultiRNAmute becomes exponential in examining all possible stabilizing and destabilizing mutations.

**Results:**

An inherent limitation in the RNAmute and MultiRNAmute programs is their ability to predict only substitution mutations, as these programs were not designed to work with deletion or insertion mutations. To address this limitation we herein develop a very fast algorithm, based on suboptimal folding solutions, to predict a predefined number of multiple point deleterious mutations as specified by the user. Depending on the user’s choice, each such set of mutations may contain combinations of deletions, insertions and substitution mutations. Additionally, we prove the hardness of predicting the most deleterious set of point mutations in structural RNAs.

**Conclusions:**

We developed a method that extends our previous MultiRNAmute method to predict insertion and deletion mutations in addition to substitutions. The additional advantage of the new method is its efficiency to find a predefined number of deleterious mutations. Our new method may be exploited by biologists and virologists prior to site-directed mutagenesis experiments, which involve indel mutations along with substitutions. For example, our method may help to investigate the change of function in an RNA virus via mutations that disrupt important motifs in its secondary structure.

## Background

The RNA molecule can be examined at several structural levels. The secondary structure of an RNA is a representation of the pattern, given an initial RNA sequence, of complementary base-pairings that are formed between the nucleic acids. Represented as a string of four letters, the sequence is a single strand that consists of the nucleotides A, C, G, and U, which are generally assumed to pair to form a secondary structure with minimum free energy. As such, the secondary structure of an RNA is experimentally accessible based on minimum free energy calculations, thus making its computational prediction a challenging but practical problem: it can be directly tested in the laboratory with minimal experimental efforts relative to, for example, RNA tertiary structure. In addition, in many cases there is a known correspondence between the secondary structure of an RNA and the molecule’s ultimate function.

In examining RNA viruses, they are known to possess unique secondary structures. The secondary structure of an RNA virus such as the Hepatitis C Virus (HCV) is mostly elongated due to the large number of base pairings that are formed, thereby lowering its free energy considerably and making the virus much more thermodynamically stable than a random RNA sequence. The typical stem-loop structure motif of an RNA virus, which consists of a long stem (a chain of consecutive base pairs) that ends in an external unpaired loop, has been experimentally observed to play a significant role in both virus replication and translation initiation. For example, in HCV, disruptive mutations were found to cause a structural change that directly led to either an alteration in virus replication [1, 2] or to a dramatic reduction in translation initiation [3].

Deleterious mutation prediction in RNAs is a sub-problem of the RNA folding prediction problem, which is fundamental in RNA bioinformatics. Thus, all tools for deleterious mutations analysis utilize methods developed for the RNA folding problem. The most common methods for RNA folding prediction in general are energy minimization methods that use dynamic programming, for example the mfold server [4], RNAstructure [5] and the ViennaRNA package and server [6, 7]. For the sub-problem considered in this work, the first publicly available methods for the analysis of deleterious mutations in RNAs were the RNAmute Java tool [8] and a web server called RDMAS [9]. Both of these methods utilize the Vienna RNA package for RNA folding prediction and are able to analyze only single point mutations in RNA sequences with applications ranging from in-silico whole-genome screening for cancer related SNPs [10], in-silico design of small RNA switches [11], studying bacterial resistance against antibiotics [12], studying the function mechanism of the spliced leader RNA [13], in addition to predicting disruptive mutations in viruses as mentioned above. To deal with multiple point deleterious mutations, the MultiRNAmute program [14] was developed, which uses an efficient method to find multiple point mutations using suboptimal folding solutions of an RNA sequence. The approach used in MultiRNAmute is based on examining a limited number of mutations, which stabilize some distant suboptimal secondary structure or/and destabilize the optimal secondary structure of the RNA sequence under consideration. Other approaches, among which the most well-known is RNAmutants [15], were also developed [16]. More recently, RNAsnp was developed [17, 18], with applications such as in studying gene variants [19] by utilizing dot plot representations that can be analyzed in a variety of ways (e.g., [20]). RNAsnp offers an efficient method to predict the effect of SNPs on local RNA secondary structure [21, 22] whereas the approaches reviewed in [16] are for global RNA secondary structure rearrangements.

A major limitation of the above described methods is that the methods are able to predict only substitution mutations, but not insertions or deletions. The suggested approach to extend the MultiRNAmute to predict deletions and insertions was briefly introduced in [23]. In addition, although the algorithm used in the MultiRNAmute program is considerably more efficient than any brute-force algorithm, it still may become exponential for sizable inputs such as sequences longer than 100-150 nts and large multiple point mutations sets. Herein, out motivation is to develop a method that predicts some predefined number (user’s input) of deleterious mutations of different types, without searching all “good” mutations as in MultiRNAmute.

The paper is organized as follow. We first prove the NP-hardness of predicting the most deleterious set of mutations in structural RNAs. We show that, even for a simplistic energy model, the associated optimization problem is NP-complete. We then describe a fast algorithm, based on the approach used in MultiRNAmute, for the prediction of a predefined number of deleterious multiple point insertion, deletion and substitution mutations. Our new method is named IndelsRNAmute, and is freely available at: https://www.cs.bgu.ac.il/~dbarash/Churkin/SCE/IndelsRNAmute/.

## Problem definition and NP-hardness

An RNA *w* is a **nucleotide sequence** of length *n* over an **alphabet** $\Sigma = \{ \mathsf{A}, \mathsf{C}, \mathsf{G}, \mathsf{U}\}$. A **secondary structure** is a set of base-pairs $S=\{(a_i,b_i)\}_i\subset [1,n]^2$ such that $a_i<b_i$, and each position is involved in at most one base pair. We consider a simple, base pair based, **energy model** where the energy of a sequence/structure pair (*w*, *S*) is given by

$$\begin{aligned} E_{w,S} := -\left| \left\{ (x,y)\in S \mid \{w_x,w_y\} \in {\mathcal {B}} \right\} \right| \text { with }{\mathcal {B}}:=\left\{ \{\mathsf{A},\mathsf{U}\},\{\mathsf{C},\mathsf{G}\},\{\mathsf{G},\mathsf{U}\}\right\} . \end{aligned}$$

Non-canonical base pairs do not contribute to the energy in the model.

For a given RNA *w*, a **mutation** is a pair $\mu =(i,b)$, expressing the choice of a new, mutated, nucleotide $b\in \Sigma -\{w_i\}$ for the position *i*. An **edit script** ${\mathcal {M}}=\{\mu _1,\ldots ,\mu _m\}$ consists of a set of mutations, each acting on a different position. Denote by $(w^{{\mathcal {M}}}$ (resp. $S^{{\mathcal {M}}}$) the **application** of an edit script ${\mathcal {M}}$ onto a sequence *w* (resp. structure *S*). Note that, when edit operations are limited to single-points mutations, one has $S^{{\mathcal {M}}} = S$.
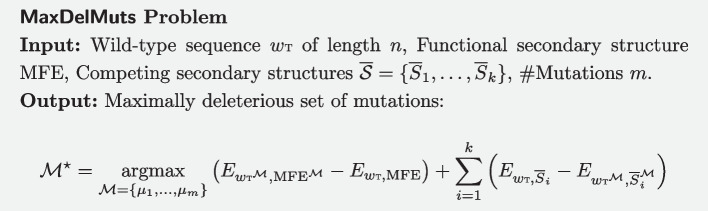


Note that the result of the $\mathop {\mathrm {argmax}}\limits$ is not affected by constant terms, so the objective can be equivalently defined as

1 $$\begin{aligned} {\mathcal {M}}^{\star } = \mathop {\mathrm {argmax}}\limits _{{\mathcal {M}}=\{\mu _1,\ldots ,\mu _m\}} E_{{w{{\textsc{{t}}}}}^{{\mathcal {M}}},\text {MFE}^{{\mathcal {M}}}} - \sum _{i=1}^k E_{{w{{\textsc{{t}}}}}^{{\mathcal {M}}},{\overline{S}}_{i}^{{\mathcal {M}}}} \end{aligned}$$

### Theorem 1

MaxDelMuts $$\in$$ NP

### Proof

Clearly, the number of ways to choose locations for *m* mutations within a sequence *w* is given by $$\left( {\begin{array}{c}n\\ m\end{array}}\right) \in \Theta (2^n)$$, while there exists exactly $3^m$ ways to assign a nucleotide content of those positions, thus the number of sets of mutations is bounded by an exponential function in *n*. Moreover, evaluating the objective function only requires the free-energy computation for $k+1$ pairs of secondary structures/mutants, which can be performed in $\Theta (n\times k)$ time.

### Theorem 2

MaxDelMuts is NP-hard.

### Proof

We first remind the MaxCoCycle problem for a graph $G=(V,E)$, which consists in finding a vertex subset $V'\subset V$ such that a maximum number of edges $E'\subset E$ see one of their ends (but not both) in $V'$.
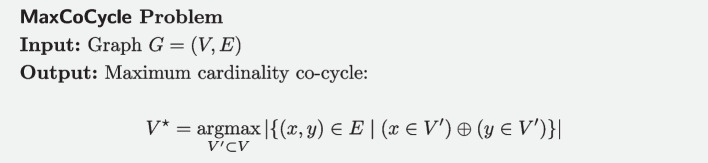


The MaxCoCycle problem was proven NP-hard by Yannakakis [24], even under the restriction of a cubic graph *G*, where all nodes have degree 3. We show that MaxCoCycle can be reduced to MaxDelMuts.

Indeed, consider an instance $G=(V,E)$ for MaxCoCycle, assuming without loss of generality that $V=[1,n]$. We build an instance of MaxDelMuts, consisting of a sequence ${w{{\textsc {{t}}}}}=\mathsf{A}^n$, a number of mutations $m=n$, a functional empty structure $\text {MFE}=\emptyset$, and a set $\overline{{\mathcal {S}}}$ of competing secondary structures, obtained by partitioning *E* into ${\mathcal {O}}(|E|)$ competing secondary structures^1^. Using the simplified expression (1), the objective function becomes:

$$\begin{aligned}{\mathcal {M}}^{\star }&= \mathop {\mathrm {argmax}}\limits _{{\mathcal {M}}} \sum _{i=1}^k - E_{{w{{\textsc {{t}}}}}^{{\mathcal {M}}},{\overline{S}}_{i}^{{\mathcal {M}}}} = \mathop {\mathrm {argmax}}\limits _{\mathcal {M}}\sum _{i=1}^k \left| \left\{ (x,y)\in {\overline{S}}_{i} \mid \{{w{{\textsc {{t}}}}}^{{\mathcal {M}}}_x,{w{{\textsc {{t}}}}}^{{\mathcal {M}}}_y\} \in {\mathcal {B}} \right\} \right| . \end{aligned}$$

Since $\overline{{\mathcal {S}}}$ represents a partition of *E*, then the expression of $E^\star$ further simplifies as:

$$\begin{aligned} {\mathcal {M}}^{\star } = \mathop {\mathrm {argmax}}\limits _{\mathcal {M}}\left| \left\{ (x,y)\in E \mid \{{w{{\textsc {{t}}}}}^{{\mathcal {M}}}_x,{w{{\textsc {{t}}}}}^{{\mathcal {M}}}_y\} \in {\mathcal {B}} \right\} \right| .\end{aligned}$$

Let us turn to the properties of ${\mathcal {M}}$ and ${w{{\textsc {{t}}}}}^{{\mathcal {M}}}$. Clearly, since $n=m$, all positions of ${w{{\textsc {{t}}}}}$ have to be mutated exactly once. Thus, after application of ${\mathcal {M}}$, there is no longer any occurrence of $\mathsf{A}$ in ${w{{\textsc {{t}}}}}^{{\mathcal {M}}}$. It follows that any base pairs contributing to the objective functions is either $\{\mathsf{G},\mathsf{C}\}$ or $\{\mathsf{G},\mathsf{U}\}$, *i.e.* a valid base pair must present exactly one occurrence of $\mathsf{G}$, thus $(\{{w{{\textsc {{t}}}}}^{{\mathcal {M}}}_x,{w{{\textsc {{t}}}}}^{{\mathcal {M}}}_y\} \in {\mathcal {B}})$ is equivalent to $(({w{{\textsc {{t}}}}}^{{\mathcal {M}}}_x = \mathsf{G}) \oplus ({w{{\textsc {{t}}}}}^{{\mathcal {M}}}_y = \mathsf{G})).$ Denoting as ${\mathcal {G}}({\mathcal {M}}) := \{ x\in [1,n]\mid {w{{\textsc {{t}}}}}^{{\mathcal {M}}}_x=\mathsf{G}\}$ the set of occurrences of $\mathsf{G}$ in ${w{{\textsc {{t}}}}}^{{\mathcal {M}}}$, one has:

$$\begin{aligned} {\mathcal {M}}^{\star }&= \mathop {\mathrm {argmax}}\limits _{\mathcal {M}}\left| \left\{ (x,y)\in E \mid \left( (x\in {\mathcal {G}}({\mathcal {M}})) \oplus (y \in {\mathcal {G}}({\mathcal {M}})\right) \right\} \right| .\end{aligned}$$

In other words, the objective value achieved by ${\mathcal {M}}$ for MaxDelMuts coincides with the objective value of ${\mathcal {G}}({\mathcal {M}})$ for MaxCoCycle.

This suggests a proof by contradiction for the optimality of ${\mathcal {G}}({\mathcal {M}}^\star )\subseteq V$ as a solution for MaxCoCycle. Denote by $\alpha$ (resp. $\beta$) the objective value of $V^{\star }$ (resp. ${\mathcal {G}}({\mathcal {M}}^{\star })$) for MaxCoCycle, and assume that $\alpha > \beta$. Then consider the edit script ${\mathcal {M}}'$, which sets all positions of $V^\star$ to $\mathsf{G}$, and all other positions to $\mathsf{C}$. ${\mathcal {M}}'$ provably achieves an objective value of $\alpha >\beta$ for MaxDelMuts. This contradicts the optimality of ${\mathcal {M}}^{\star }$, and one concludes that $\alpha =\beta$, i.e. ${\mathcal {G}}({\mathcal {M}}^\star )$ represents a (co-)optimal solution of MaxCoCycle. Thus any polynomial algorithm for solving MaxDelMuts, coupled with a linear time computation of ${\mathcal {G}}({\mathcal {M}}^\star )$, would provide an exact polynomial algorithm for the NP-hard MaxCoCycle. Therefore, MaxDelMuts is NP-hard.

## Methods

Similar to the MultiRNAmute method, the IndelRNAmute method uses suboptimal secondary structures as a starting point. The motivation behind this decision is to start with some distant (from optimal structure) suboptimal structures and to convert such suboptimal structures to an optimal one by introducing “wise” mutations, which stabilize the stems of the suboptimal structure and destabilize the stems of the optimal one.

The mutation analysis algorithm consists of several steps. First, given an input sequence with several input parameters, the Minimum Free-Energy (MFE) and a set of suboptimal secondary structures are calculated using the RNAfold and RNAsubopt programs from the Vienna RNA package [6], followed by a filtering step to reduce the number of suboptimal structures. Next, for each optimal and suboptimal structure, stems are identified and used for selecting “good” single-point mutations of several types: insertions, deletions and substitutions, depending on the user’s choice.
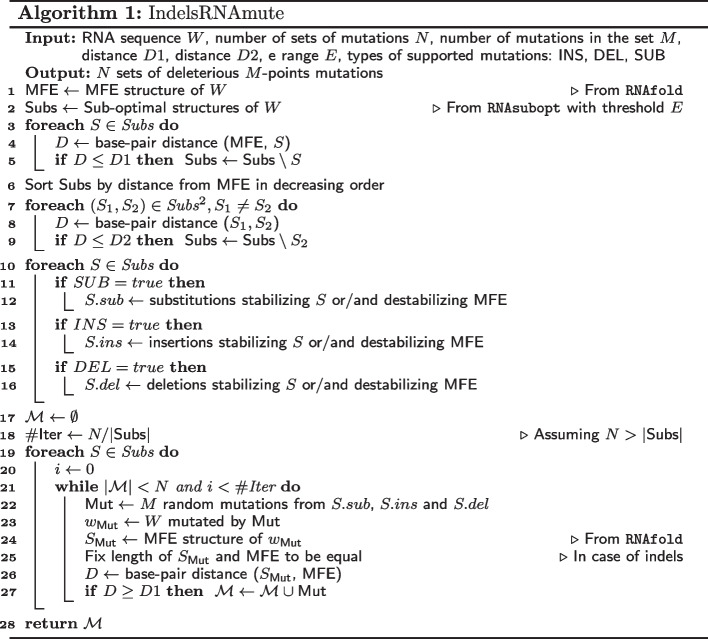


Finally, these single-point mutations are combined together to form deleterious multiple-point mutations for the output. A summary of the algorithm is shown in Algorithm 1. We expand on each step of the method in the following sub-sections.

### Input parameters

The parameters of the method include:

- RNA sequence field (*S*)—the maximum sequence length allowed in our application is 1000 bases;
- dist 1 ($D_1$)—this distance parameter is used for filtering suboptimal solutions that are close to the optimal solution. The suggested value to use is around 30% of the RNA sequence length;
- dist 2 ($D_2$)—this distance parameter is used for filtering suboptimal solutions that are close to each other. The suggested value to use is around 30% of the RNA sequence length;
- e range (*E*)—this energy parameter is used in the *RNAsubopt* program to calculate the suboptimal structures within a range of kcals/mol of the mfe. The suggested value is around 15% of the RNA sequence length;
- #Point mutations (*M*)—number of allowed point mutations in RNA (one M-point mutation set);
- #Results (*N*)—number of *M*-point mutations in the output;
- Type of mutations (SUM, INS, DEL)—the user may choose to allow insertions, deletions and substitutions in the M-point mutation set;
- Open Prev Run—The application saves the results in a file, allowing to open previous runs without running the application again;
- Open Run—The user may save the results and insert them later in the GUI.

### Optimal and suboptimal structures calculation

At the initial step, after starting the calculation by pressing “Start” in the GUI, the program calculates the dot-bracket representations of the optimal and suboptimal secondary structures of the provided RNA sequence. The optimal structure is calculated using RNAfold and the suboptimal structures are calculated using RNAsubopt with parameter *e* from the GUI. Both routines are available in the Vienna RNA package [6].

### Filtering suboptimal secondary structures

Running RNAsubopt may lead to a huge number of, largely redundant, suboptimal folding solutions. In order to consider a small and diverse set of suboptimal structures, distant from the optimal structure, we use two filters. The first filter removes all suboptimal structures that are similar to the optimal one using *dist*1 input parameter as a distance threshold. After the first filter the suboptimal structures are sorted by their distance from the optimal structure. The second filter removes suboptimal structures that are close to each other.

Herein for each set of similar structures we proceed with only one representative that is the most distant from the optimal structure and also distant from all representatives of other sets. As an example, Table 1 shows structures generated for an artificial RNA sequence:

**Table 1:** Optimal and suboptimal structures of the artificial RNA sequence after filtering

Structure	Dot-bracket representation	Distance
opt	(((((((.((((((...((((((....))))))....))))))...(((((.(((((....))))).)))))..((((...)))).)))))))	0
sub1	.........(((.....)))..((((...(((((.((..(((....(((((.(((((....))))).)))))..)))..))))))).))))..	43
sub2	(((((((.((((((((((((((((.....(((((((..........))).))))))))...)))).)))))...........))).)))))))	39
sub3	(((((((.(((.((......(((...((..(((.......)))..))...)))((((.(((((.....))))).)))).)).))).)))))))	39
sub4	(((((..((.(((.(((((((((....))))))......((......))....((((.(((((.....))))).)))).)))))).)))))))	36
sub5	.((((((.(((.((...((((((....))))))...((.(((((...((.....))...))))).))((((...)))).)).))).)))))).	35
sub6	........((((((...((((((....))))))....))))))..((((....((((.(((((.....))))).)))).....))))......	34

CCGGAAGAGGGGGACAACCCGGGGAAACUCGGGCUAAUCCCCCAUGU

GGACCCGCCCCUUGGGGUGUGUCCAAAGGGCUUUGCCCGCUUCCGG

The table contains the optimal secondary structure and 6 suboptimal structures that passed the filtering stage with dist1 and dist2 thresholds = 30 and *e* = 15. The first row in the table corresponds to the optimal structure and the six rows below correspond to the suboptimal structures. The last column in the table shows the base-pair distance of the structure from the optimal structure. If more distant suboptimal structures are required, the *e* parameter in the GUI should be increased.

### Collecting candidates for deleterious mutations

For each suboptimal structure that survived the filtering, we find mutations (insertions, deletions and substitutions depending on the user’s choice) that may potentially convert the optimal secondary structure to a suboptimal one. To perform this task, we first calculate the start and end positions of all stems in the optimal and all suboptimal structures. For instance, the secondary structure ((((..(((....))).)))) has two stems, with start/end at positions (1, 21)/(4, 18) and (7, 16)/(9, 14), respectively.

Next, we collect the mutations that stabilize the stems of the suboptimal structure and destabilize the stems of the optimal structure. The program searches for “good” places (indices) in the sequence for potential deleterious mutations. The “good” places for mutations are between stems of the suboptimal structure and in the middle of the stems of the optimal structure. This is true for substitutions, insertion and deletions. In the case of insertions that destabilize the optimal structure, it is possible to insert an exponential (in the size of M-mutation set) number of combinations of insertions in each index of the stem. To solve this problem we allow to insert only one mutation somewhere in the middle of each stem of the optimal structure. This is sufficient for the destabilization of the stem.

#### Example 1

Deleterious deletions:

GAGUGUCGACUCCGCC - RNA wildtype sequence

((((....)))).... - Optimal structure

..(.(.((....)))) - Suboptimal structure

In the example above the “good” indices for deletions are 4 and 6. Deletions U4 and U6 stabilize (elongate) the stems of the suboptimal structure, while mutation U4 also destabilizes (shortens) the single stem of the optimal structure. By introducing two point mutation U4-U6 into the wild type RNA sequence we obtain the following result:

GAGGCGACUCCGCC - RNA wildtype sequence

(((....))).... - Optimal structure

..((((....)))) - Suboptimal structure

We can clearly see from the example that mutation U4-U6, consisting of two deletions, converts the suboptimal structure to become more stable than the optimal one.

#### Example 2

Deleterious insertions and substitutions:

GAGGGUCGCCUCCGCGC - RNA wildtype sequence

((((....))))..... - Optimal structure

..(.(.((....))).) - Suboptimal structure

In this example, one of the “good” indices for substitution is 4 and one of the “good” indices for insertions is 15 (insertion between two stems from the narrow side). Substitution G4C connects two stems of the suboptimal structure and shortens one stem of the optimal structure. Insertion 15A connects two stems of the suboptimal structure. Finally, by introducing the two mutations G4C-15A into the wildtype RNA sequence we obtain the following result:

GAGCGUCGCCUCCGACGC - RNA wildtype sequence

(((......)))...... - Optimal structure

..((((((....)))))) - Suboptimal structure

### Calculation of M-point mutation sets

At this stage, the program combines deletions, insertions and substitutions up to *N* sets of *M* mutations. The algorithm is implemented in a recursive way that searches all possible combinations of all types of mutations found in the previous stage, but stops after reaching *N* mutations or all possible combinations of *M*-sets (if *N* is very large). For sequences longer than 150 bases, and values of *M* greater than 3, the number of all possible *M*-sets may be very large, much larger than *N* provided by the user.

Practically, it is sufficient to find a small amount of deleterious mutations (no more than 100) for laboratory experiments. In order to obtain a diversity of mutation types in the output, the algorithm combines single-point mutations randomly by choosing the calculation path through mutation types in a random way. To add to the diversity in the output, the algorithm uses all available diverse suboptimal structures for mutation analysis. For example, if the *N* provided by the user is 100 and the filtering stage produces 5 suboptimal structures, the algorithm will limit itself to 20 random deleterious *M*-point mutation sets for each suboptimal structure. The deleterious nature of each *M*-point mutation is validated by checking that the mutation structure is distant enough from the structure of the wild type RNA sequence.

## Results

**Fig. 1:**
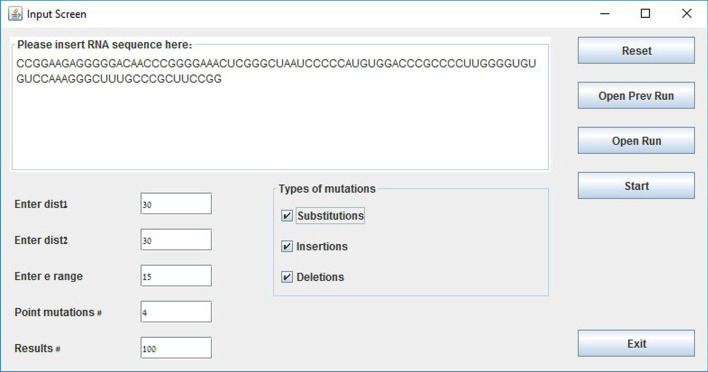
Input screen of IndelsRNAmute with an example input sequence and other parameters for the results shown in Fig. 2

**Fig. 2:**
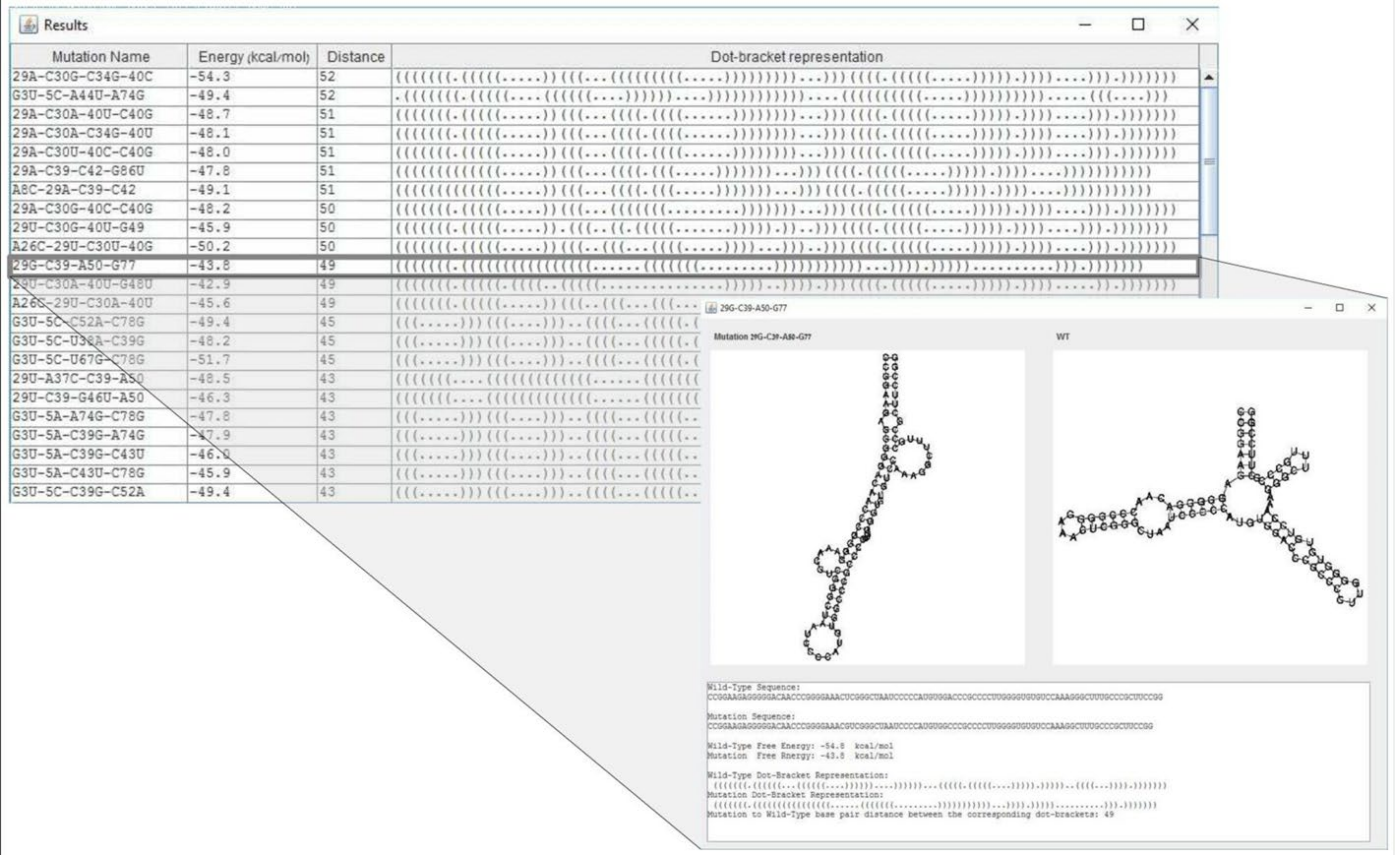
Typical output of IndelsRNAmute and detailed structural analysis of mutation $29G-C39-A50-G77$

The input screen of IndelsRNAmute with an example sequence and input parameters is shown in Fig. 1. A typical output of IndelsRNAmute is shown in Fig. 2. The resultant parameters for the sequence discussed in Sect. 3.3 are $dist1=30$, $dist2=30$, $e=15$, $N=100$, $M=4$ and all three types of mutations. The output lists up to *N* *M*-point mutations, sorted by the distance of their structure from the wildtype structure. The most deleterious mutations are listed first. Each row in the table includes the name of mutation, free energy, distance from wildtype RNA and the dot-bracket representation of its structure.

### Interactive features

A user may further investigate a given mutation, by pressing on some row in the table to see more information about a specific mutation. For example, selecting the mutation $29G-C39-A50-G77$ will open the screen shown in Fig. 2. The structure of this mutation was obtained from the second suboptimal structure in Table 1 by one insertion and three deletions. The insertion 29G and deletions C39 and G77 destabilize stems in the optimal structure, while deletion A50 both stabilizes the suboptimal structure by connecting two stems and destabilizes the stem of the optimal structure.

### Analysis of mutations sets for random sequences

**Fig. 3:**
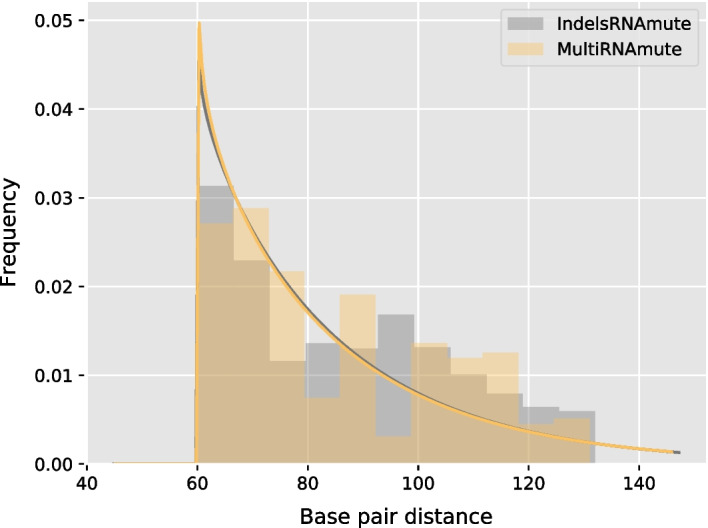
Distributions of base-pair distance to the WT MFE of mutations sets ($M=5$) produced for random sequences of length 200 nts

**Fig. 4:**
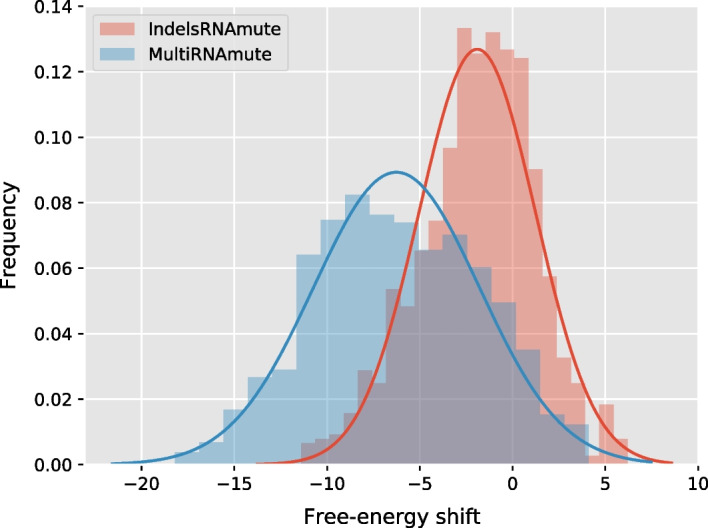
Distributions of energy distance to the WT MFE of mutations sets ($M=5$) produced for random sequences of length 200 nts

The importance of considering indels in the prediction of deleterious sets of mutations is illustrated by Fig. 3 and Fig. 4. In this analysis, 100 sets of $M=5$ deleterious mutations were predicted for 10 random sequences of length 200 nts, respectively, in the presence and absence of support for indels.

As can be seen in Fig. 3, mutations in presence/absence of indels are equally deleterious, and induce distributions whose exponential fitted curves are virtually indistinguishable. However, as shown in Fig. 4, mutations sets including indels retain comparable free-energy as the wild type, while substitutions appear to induce a drastic decrease of the free-energy.

We interpret these results as indicative of the fact that mutations sets including indels, predicted by IndelsRNAmute, are much more geared towards the identification of deleterious sets of mutations, rather than a mere optimization of the thermodynamic stability of alternative structures. This interpretation suggests more realistic sets of mutations being produced by IndelsRNAmute. Indeed, due to kinetics effects, alternative structures associated with extreme shifts in MFE may not be reachable within folding landscapes in time comparable to adverse processes such as RNA degradation.

## Conclusion

We present a method called IndelsRNAmute that extends our MultiRNAmute method to predict insertion and deletion mutations in addition to substitutions. The additional advantage of the new method is its efficiency to find a predefined number of deleterious mutations. The running time of MultiRNAmute depends on the number of possible deleterious mutations, which may be very large and depend exponentially on number of mutations in the multiple-point mutation set, while the running time of IndelsRNAmute depends on *N* and only depends linearly on *M*. For example, for the same input, MultiRNAmute may run more than an hour predicting only substitutions, while IndelsRNAmute predicts 100 “good” mutations in a few seconds, and depending on the user’s choice may include insertions, deletions and substitutions. We do not compare running times of MultiRNAmute with IndelsRNAmute in a quantitative manner because the fast running time of the new method is achieved only by limiting the size of the output. Not limiting the size of the output of the new method will cause it to run slower than MultiRNAmute because more types of mutations and their combinations are taken into account. Obviously, when limiting the size of the output, IndelsRNAmute would run faster.

**Fig. 5:**
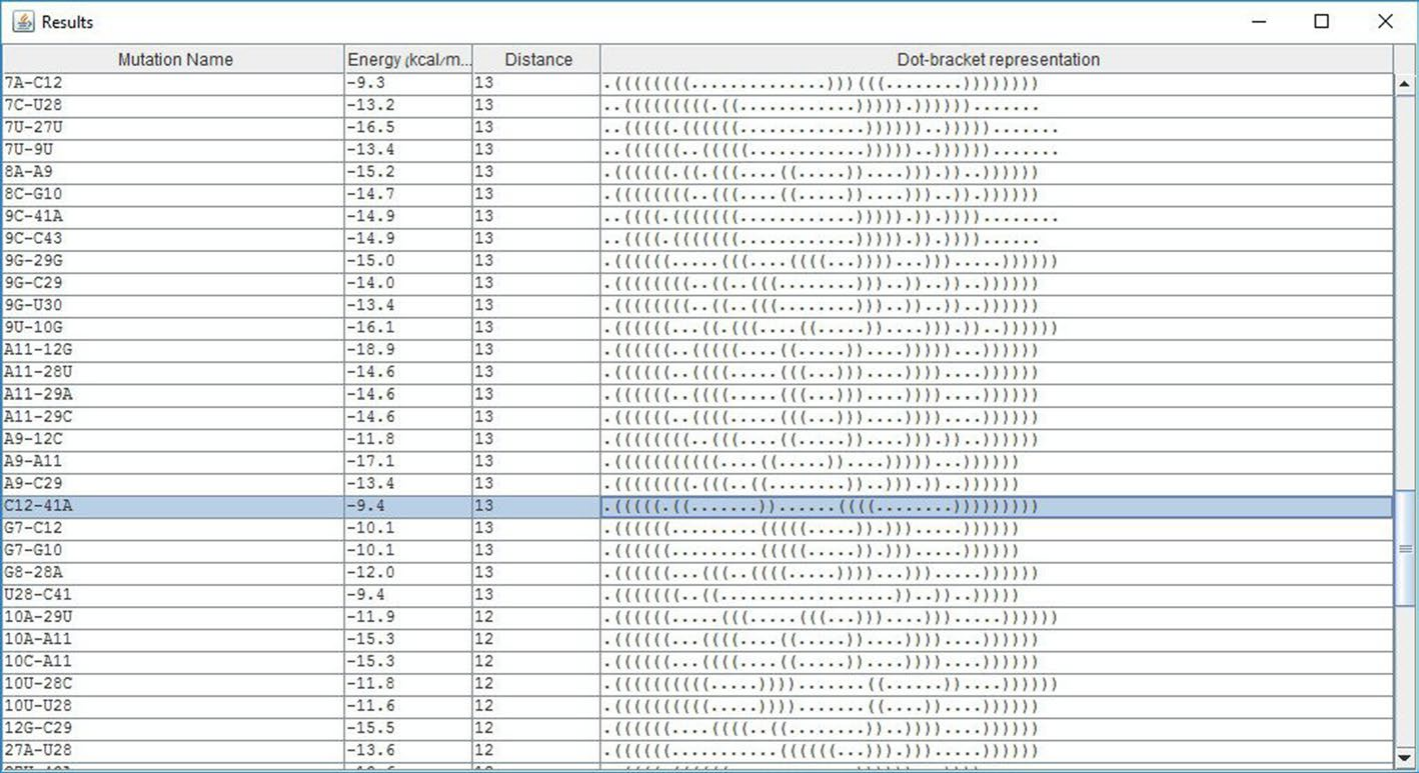
List of mutations screen of IndelsRNAmute, for the case of 2-point indel mutations in the 5BSL3.2 wild-type

**Fig. 6:**
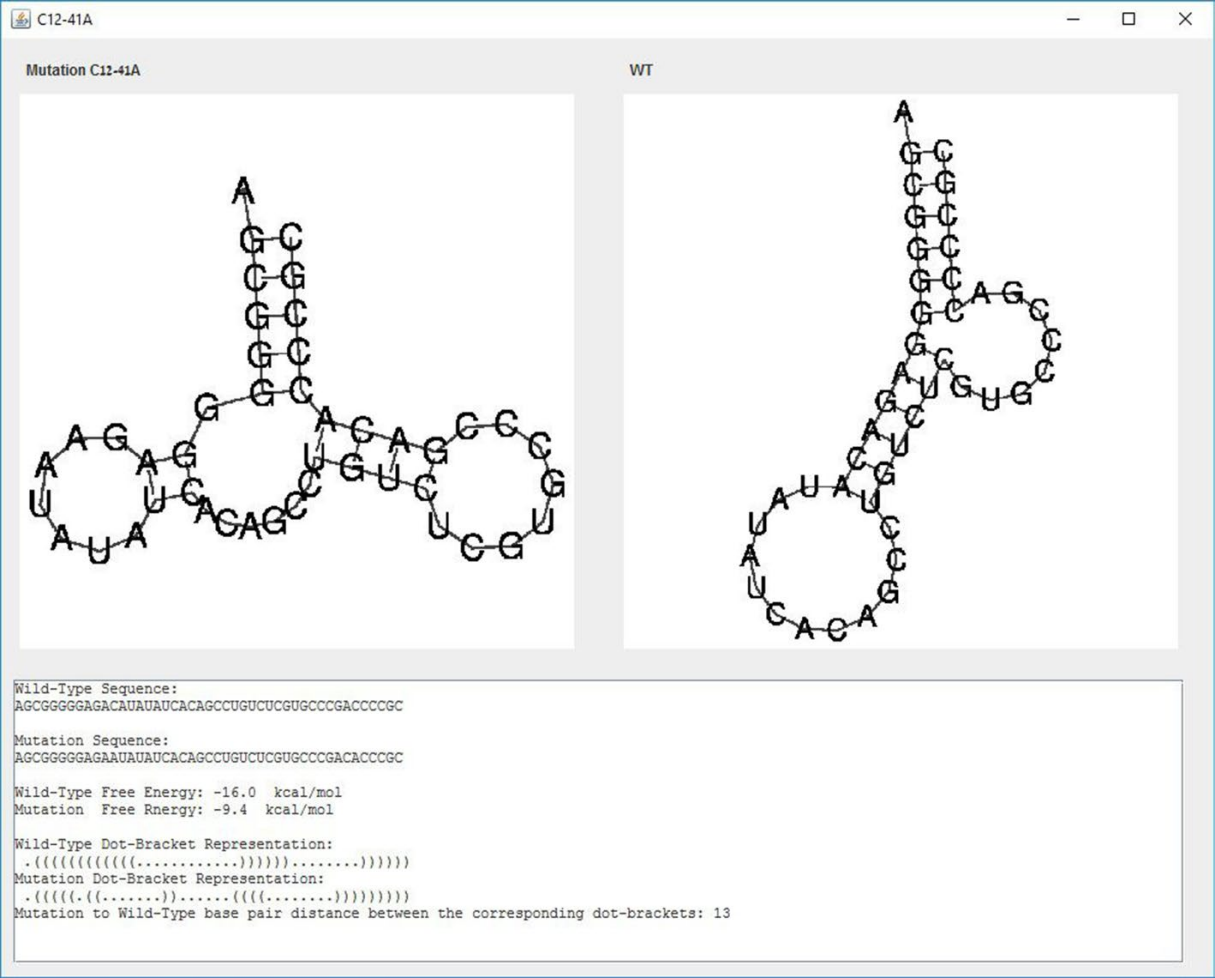
Output screen of one of the rearranging 2-point indel mutations in the 5BSL3.2

All our mutation prediction methods were shown practical in predicting deleterious mutations in the P5abc subdomain of the *Tetrahymena thermophila* group 1 intron ribozyme, and in the 5BSL3.2 sequence of a subgenomic HCV replicon [2]. Fig. 5 illustrates potential deleterious 2-point indel mutations in the 5BSL3.2 sequence of a subgenomic HCV replicon, in addition to 2-points deleterious substitutions, which may be predicted by both IndelsRNAmute and MultiRNAmute. One such experimentally approved deleterious 2-point substitution was reported in [14], and one of the good canditates for deleterious 2-point indel mutations (C12-41A) predicted by IndelsRNAmute is illustrated in Fig. 6.

**Fig. 7:**
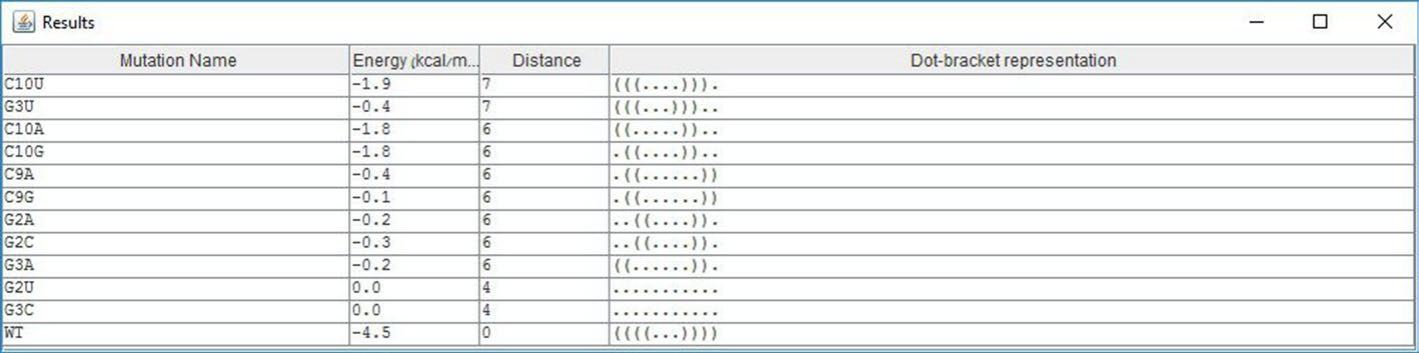
Output screen of the rearranging 1-point substitutions in GGGGAAACCC sequence, using MultiRNAmute method or IndelsRNAmute method with “Substitutions” option

**Fig. 8:**
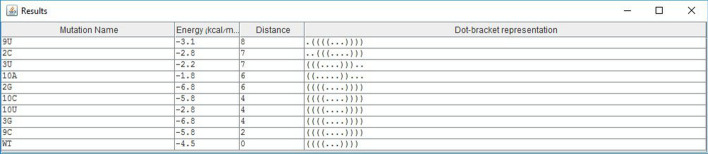
Output screen of the rearranging 1-point insertions in GGGGAAACCC sequence, using IndelsRNAmute method with “Insertions” option

**Fig. 9:**
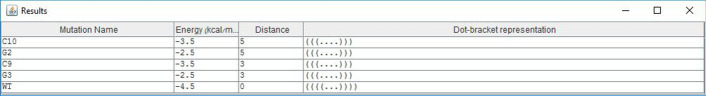
Output screen of the rearranging 1-point deletions in GGGGAAACCC sequence, using IndelsRNAmute method with “Deletions” option

In order to show the potential difference between IndelsRNAmute and the MultiRNAmute method, we ran both methods on a very short sequence GGGGAAACCCC and with only one point mutation. Figure 7 shows the output of the MultiRNAmute method that contains only substitution mutations. Using the IndelsRNAmute method we may obtain the results shown in Fig. 7 by selecting the “Substitutions” option in the input screen. In addition, we can obtain the results shown in Figs. 8 and 9 by selecting the “Insertions” and “Deletions” options, respectively.

In future work we plan to implement *k*-medoids clustering, using medoids (centroids) as set representatives instead of our current filtering. A significant concern to overcome with such a clustering method could be that finding the optimal *k* is time consuming and the user will have to provide *k* as an additional parameter in the GUI and some “good” suboptimal structures may be missed. The advantages in pursuing this clustering strategy is that it will explain filtering better in terms of thermodynamics. In all distance calculations in our application we use linear base-pair distance for efficiency, but the method can be easily adapted to work with any other distance, like Hamming distance or tree edit distance as possible extensions.

## Data Availability

All data generated or analysed during this study are included in this published article or may be easily produced by the following tool: https://www.cs.bgu.ac.il/~dbarash/Churkin/SCE/IndelsRNAmute/.

## Footnotes

1 Note that, if crossing pairs (*aka* pseudoknots) are allowed, the Vizing theorem implies that $\overline{{\mathcal {S}}}$ can be reduced in polynomial time to 3 structures, although this observation bears no consequence on the hardness of the problem.
